# Automation of literature screening using machine learning in medical evidence synthesis: a diagnostic test accuracy systematic review protocol

**DOI:** 10.1186/s13643-021-01881-5

**Published:** 2022-01-15

**Authors:** Yuelun Zhang, Siyu Liang, Yunying Feng, Qing Wang, Feng Sun, Shi Chen, Yiying Yang, Xin He, Huijuan Zhu, Hui Pan

**Affiliations:** 1 Medical Research Center, Peking Union Medical College Hospital, Chinese Academy of Medical Sciences and Peking Union Medical College, Beijing, China; 2 Department of Endocrinology, Peking Union Medical College Hospital, Chinese Academy of Medical Sciences and Peking Union Medical College, 1 Shuaifuyuan, Dongcheng District, Beijing, China; 3 Eight-year Program of Clinical Medicine, Peking Union Medical College Hospital, Chinese Academy of Medical Sciences and Peking Union Medical College, Beijing, China; 4 Research Institute of Information and Technology, Tsinghua University, Beijing, China; 5 Department of Epidemiology and Biostatistics, School of Public Health, Peking University Health Science Center, Beijing, China

**Keywords:** Evidence-based practice, Artificial intelligence, Natural language process, Protocol, Systematic review, Diagnostic test accuracy

## Abstract

**Background:**

Systematic review is an indispensable tool for optimal evidence collection and evaluation in evidence-based medicine. However, the explosive increase of the original literatures makes it difficult to accomplish critical appraisal and regular update. Artificial intelligence (AI) algorithms have been applied to automate the literature screening procedure in medical systematic reviews. In these studies, different algorithms were used and results with great variance were reported. It is therefore imperative to systematically review and analyse the developed automatic methods for literature screening and their effectiveness reported in current studies.

**Methods:**

An electronic search will be conducted using PubMed, Embase, ACM Digital Library, and IEEE Xplore Digital Library databases, as well as literatures found through supplementary search in Google scholar, on automatic methods for literature screening in systematic reviews. Two reviewers will independently conduct the primary screening of the articles and data extraction, in which nonconformities will be solved by discussion with a methodologist. Data will be extracted from eligible studies, including the basic characteristics of study, the information of training set and validation set, and the function and performance of AI algorithms, and summarised in a table. The risk of bias and applicability of the eligible studies will be assessed by the two reviewers independently based on Quality Assessment of Diagnostic Accuracy Studies (QUADAS-2). Quantitative analyses, if appropriate, will also be performed.

**Discussion:**

Automating systematic review process is of great help in reducing workload in evidence-based practice. Results from this systematic review will provide essential summary of the current development of AI algorithms for automatic literature screening in medical evidence synthesis and help to inspire further studies in this field.

**Systematic review registration:**

PROSPERO CRD42020170815 (28 April 2020).

**Supplementary Information:**

The online version contains supplementary material available at 10.1186/s13643-021-01881-5.

## Background

Systematic reviews synthesise the results of multiple original publications to provide clinicians with comprehensive knowledge and current optimal evidence in answering certain research questions. The major steps of a systematic review are defining a structured review question, developing inclusion criteria, searching in the databases, screening for relevant studies, collecting data from relevant studies, assessing the risk of bias critically, undertaking meta-analyses where appropriate, and assessing reporting biases [1–3]. A systematic review aims to provide a complete, exhaustive summary of current literature relevant to a research question with an objective and transparent approach. In the light of these characteristics, systematic reviews, in particular those combining high quality evidence, which used to be at the very top of the medical evidence pyramid [4] and now become regarded as an indispensable tool for evidence viewing [5], are widely used by reviewers in the practice of evidence-based medicine.

However, conducting systematic reviews for clinical decision making is time-consuming and labour-intensive, as the reviewers are supposed to perform a thorough search to identify any literatures that may be relevant, read through all abstracts of retrieved literatures, and identify the potential candidates for further full-text screening [6]. For original researches, the median time from the publication to their first inclusion in a systematic review ranged from 2.5 to 6.5 years [7]. It usually takes over a year to publish a systematic review from the time of literature search [8]. However, with advances in clinical research, this evidence and systematic review conclusions it generates may be out of date within several years. With the explosive increase of original research articles, reviewers have found difficulty identifying most relevant evidence in time, let alone updating systematic reviews periodically [9]. Therefore, researchers are exploring automatic methods to improve the efficacy of evidence synthesis while reducing the workload of systematic reviews.

Recent progresses in computer science show a promising future that more intelligent works can be accomplished with the aid of automatic technologies, such as pattern recognition and machine learning (ML). Being seen as a subset of artificial intelligence (AI), ML utilises algorithms to build mathematical models based on training data in order to make predictions or decisions without being explicitly programmed [10]. Various ML studies have been introduced in the medical field, such as diagnosis, prognosis, genetic analysis, and drug screening, to support clinical decision making [11–14]. When it comes to automatic methods for systematic reviews, models for automatic literature screening have been explored to reduce repetitive work and save time for reviewers [15, 16].

To date, limited research has been focused on automatic methods used for biomedical literature screening in systematic review process. Automated literature classification systems [17] or hybrid relevance rating models [18] were tested in specific datasets, yet further extension of review datasets and performance improvement are required. To address this gap in knowledge, this article describes the protocol for a systematic review aiming at summarising existing automatic methods to screen relevant biomedical literature in the systematic review process, and evaluating the accuracy of the AI tools.

## Methods

### Objectives

The primary objective of this review is to assess the diagnostic accuracy of AI algorithms (index test) compared with gold-standard human investigators (reference standard) for screening relevant literatures from original literatures identified by electronic search in systematic review. The secondary objective of this review is to describe the time and work saved by AI algorithms in literature screening. Additionally, we plan to conduct subgroup analyses to explore the potential factors that associate with the accuracy of AI algorithms.

### Study registration

We prepared this protocol following the Preferred Reporting Items for Systematic Review and Meta-Analysis Protocols (PRISMA-P) [19]. This systematic review has been registered on PROSPERO (Registration number: CRD42020170815, 28 April 2020).

### Review question

Our review question was refined using PRISMA-DTA framework, as detailed in Table 1. In this systematic review, “literatures” refer to the subjects of the diagnostic test (the “participants” in Table 1), and “studies” refer to the studies included in our review.

**Table 1:** Review question

Item	Description
“Participants”*	Original publications and literatures identified by electronic literature search
Index test	Automatic literature screening models using artificial intelligence algorithms
Reference standard	Traditional literature screening by human investigators
Outcome	Primary outcome: diagnostic accuracy, measured by sensitivity, specificity, precision, NPV, PPV, NLR, PLR, DOR, F-measure, accuracy, and AUC of automatic literature screening models Secondary outcomes: labour and time saving, mainly evaluated by the percentage of retrieved literatures that the reviewers do not have to read (because they have been screened out by the automatic literature screening models)

*Abbreviations: AUC*, area under curve; *DOR*, diagnostic odds ratio; *NLR*, negative likelihood ratio; *NPV*, negative predictive value; *PLR*, positive likelihood ratio; *PPV*, positive predictive value

*The “participants” in our review refer to the original publications and literatures identified in a systematic literature search, rather than human participants or patients in traditional systematic reviews

### Inclusion and exclusion criteria

We will include studies in medical research that reported a structured study question, described the source of the training or validation sets, developed or employed AI models for automatic literature screening, and used the screening results from human investigators as the reference standard.

We will exclude traditional clinical studies in human participants, editorials, commentaries, or other non-original reports. Pure methodological studies in AI algorithms without application in evidence synthesis will be excluded as well.

### Information source and search strategy

An experienced methodologist will conduct searches in major public electronic medical and computer science databases, including PubMed, Embase, ACM Digital Library, and IEEE Xplore Digital Library, for publications ranged from January 2000 to present. We set this time range because to the best of our knowledge, AI algorithms prior to 2000 are unlikely to be applicable in evidence synthesis [20]. In addition to the literature search, we will also find more relevant studies through checking the reference lists of included studies identified by electronic search. Related abstracts and preprints will be searched in Google scholar. There are no language restrictions in searches. We will use free text words, MeSH/EMTREE terms, IEEE Terms, INSPEC Terms, and ACM Computing Classification System to develop strategies related to three major concepts: systematic review, literature screening, and AI. Multiple synonyms for each concept will be incorporated into the search. The Systematic Review Toolbox (http://systematicreviewtools.com/) will also be utilised to detect potential automation methods in medical research evidence synthesis. Detailed search strategy used in PubMed is shown in Supplementary Material 1.

### Study selection

Literatures with titles and abstracts from online electronic databases will be downloaded and imported into EndNote X9.3.2 software (Thomson Reuters, Toronto, Ontario, Canada) for further process after removing duplications.

All studies will be screened independently by 2 authors based on the titles and abstracts. Those which do not meet the inclusion criteria will be excluded with specific reasons. Disagreements will be solved by discussion with a methodologist if necessary. After the initial screening, the full texts of the potentially relevant studies will be independently reviewed by the two authors to make decisions on final inclusions. Conflicts will be resolved in the same way as they were initially screened. Excluded studies will be listed and noted according to PRISMA-DTA flowchart.

### Data collection

A data collection form will be used for information extraction. Data from the eligible studies will be independently extracted and verified by two investigators. Disagreements will be resolved through discussion and consultation with the original publication. We will also try to contact the authors to collect the missing data. If one study did not report detailed accuracy data or did not provide enough data that are essential to calculate the accuracy data, this study will be omitted from the quantitative data synthesis.

The following data will be extracted from the original studies: characteristics of study, information of training set and validation set, and the function and performance of AI algorithms. The definitions of variables in data extraction are shown in Table 2.

**Table 2:** Definitions of variables in data extraction

Variable	Definitions
Study characteristics	
Year	Year of publication
Authors	Last name of authors
Study type	Article, abstract, or systematic review
Journal, conference	Name of journal or conference
Training set information	
Training set	Name of dataset used for training
Area	General medicine, detailed disease, or specific intervention
Source	Name of electronic databases searched for building training set
Time range	Time range of training set
Type of publication	Abstract, or full-text
Number of all literatures	Number of all literatures in training set
Number of included literatures	Number of included literatures identified by the step of screening in training set
Training method	Supervised, semi-supervised, or unsupervised
Validation set information	
Validation set	Name of dataset used for validation
Area	General, disease, or intervention
Source	Name of electronic database searched for building validation set
Time range	Time range of validation set
Type of publication	Abstract, or full-text
Number of all literatures	Number of all literatures in validation set
Number of included literatures	Number of included literatures identified by the step of screening in validation set
Golden standard	Process of screening by human investigators
AI algorithm information	
Model name	Name of model
Model type	Classification, regression, ranking, or others
Model performance	Including but not limited to sensitivity, specificity, precision, NPV, PPV, NLR, PLR, DOR, F-measure, accuracy, and AUC
Cost saving	Decreased number of screened literatures by human investigators

*Abbreviations: AUC*, area under curve; *DOR*, diagnostic odds ratio; *NLR*, negative likelihood ratio; *NPV*, negative predictive value; *PLR*, positive likelihood ratio; *PPV*, positive predictive value

### Risk of bias assessment, applicability, and levels of evidence

Two authors will independently assess risk of bias and applicability with a checklist based on Quality Assessment of Diagnostic Accuracy Studies (QUADAS-2) [21]. The QUADAS-2 contains 4 domains, respectively regarding patient selection, index test, reference standard, and flow and timing risk of bias. The risk of bias is classified as “low”, “high”, or “unclear”. Studies with high risk of bias will be excluded in the sensitivity analysis.

In this systematic review, the “participants” are literatures rather than human subjects. The index test is AI model used for automatic literature screening. Therefore, we will slightly revise the QUADAS-2 to fit our research context (Table 3). We deleted one signal question in the QUADAS-2 “was there an appropriate interval between index test and reference standard”. The purpose of this signal question in the original version of the QUADAS-2 is to judge the bias caused by the change of disease status between the index test and the reference test. The “disease status”, or the final inclusion status of one literature in our research context, will not change; thus, there are no such concerns.

**Table 3:** The revised QUADAS-2 tool for risk of bias assessment

Domains	Signal questions	Answers
“Patient” (literature) Selection	**Risk of bias**	
	Was a consecutive or random sample of literatures enrolled	Yes/no/unclear
	Was a case-control design avoided	Yes/no/unclear
	Did the study avoid inappropriate exclusions	Yes/no/unclear
	Could the selection of literatures have introduced bias	Low/high/unclear risk
	**Concerns regarding applicability**	
	Is there concern that the included literatures do not match the review question	Low/high/unclear risk
Index test (AI algorithms in literature screening)	**Risk of bias**	
	Were the index test results interpreted without knowledge of the results of the reference standard	Yes/no/unclear
	If a threshold was used, was it pre-specified	Yes/no/unclear
	Could the conduct or interpretation of the index test have introduced bias	Low/high/unclear risk
	**Concerns regarding applicability**	
	Is there concern that the index test, its conduct, or interpretation differ from the review question	Low/high/unclear risk
Reference standard (results of screening by human investigators)	**Risk of bias**	
	Is the reference standard likely to correctly classify the target condition	Yes/no/unclear
	Were the reference standard results interpreted without knowledge of the results of the index test	Yes/no/unclear
	Could the reference standard, its conduct, or its interpretation have introduced bias	Low/high/unclear risk
	**Concerns regarding applicability**	
	Is there concern that the target condition as defined by the reference standard does not match the review question	Low/high/unclear risk
Flow and timing	**Risk of bias**	
	Did all literatures receive a reference standard	Yes/no/unclear
	Did literatures receive the same reference standard	Yes/no/unclear
	Were all literatures included in the analysis	Yes/no/unclear
	Could the literature flow have introduced bias	Low/high/unclear risk

The levels of the evidence body will be evaluated by the Grading of Recommendations, Assessment, Development and Evaluations (GRADE) framework [22].

### Diagnostic accuracy measures

We will extract the data of per study in a two-by-two contingency table from the formal publication text, appendices, or by contacting the main authors to collect sensitivity, specificity, precision, negative predictive value (NPV), positive predictive value (PPV), negative likelihood ratio (NLR), positive likelihood ratio (PLR), diagnostic odds ratios (DOR), F-measure, and accuracy with 95% CI. If the outcomes cannot be formulated in a two-by-two contingency table, we will extract the reported performance data. If possible, we will also assess the area under the curve (AUC), as the two-by-two contingency table may not be available in some scenarios.

### Qualitative and quantitative synthesis of results

We will qualitatively describe the application of AI in literature screening and evaluate and compare the accuracy of the AI tools. If there were adequate details and homogeneous data for the quantitative meta-analysis, we will combine the accuracy of AI algorithms in literature screening using the random-effects Rutter-Gatsonis hierarchical summarised receiver operating characteristic curve (HSROC) model which was recommended by the Cochrane Collaboration for combining the evidence for diagnostic accuracy [23]. The effect of threshold will be incorporated in the model in which heterogeneous thresholds among different studies will be allowed. The combined point estimates of accuracy will be retrieved from the summarised receiver operating characteristic curve (ROC).

Subgroup analyses and meta-regression will be used to explore the between-study heterogeneity. We will explore the following predefined sources of heterogeneity: (1) AI algorithm type, (2) study area of validation set (targeted specific diseases, interventions, or a general area), (3) searched electronic databases (PubMed, EMBASE, or others), and (4) proportion of eligible to original studies (the number of eligible literature identified in the screening step divided by the number of original literature identified during the electronic search). Furthermore, we will analyse the possible sources of heterogeneity from both dataset and methodological perspectives in HSROC as covariates following the recommendations from the Cochrane Handbook for Diagnostic Tests Review [23]. We regarded the factor as a source of heterogeneity if the coefficient of the covariate in the HSROC model was statistically significant. We will not evaluate the reporting bias (e.g. publication bias) since the hypothesis underlying the commonly used methods, such as funnel plot or Egger’s test, may not be satisfied in our research context. Data were analysed using R software, version 4.0.2 (R Foundation for Statistical Computing, Vienna, Austria) with two-tailed probability of type I error of 0.05 (*α*=0.05).

## Discussion

Systematic review has developed rapidly within the last decades and plays a key role in enabling the spread of evidence-based practice. Systematic review, though costing less than primary research in money expenditure, is still time-consuming and labour-intensive. Conducting systematic review begins with electronic database searching for a specific research question, then at least two reviewers read each abstract of retrieved records to identify potential candidate literatures for full-text screening. Only 2.9% retrieved records are relevant and included in the final synthesis on average [24]; typically, reviewers have to find the proverbial needle in the haystack of irrelevant titles and abstracts. Computational scientists have developed various algorithms for automatic literature screening. Developing an automatic literature screening instrument will be source-saving and improve the quality of systematic review by liberating reviewers from repetitive work. In this systematic review, we aim to describe and evaluate the development process and algorithms used in various AI literature screening systems, in order to build a pipeline for the update of existing tools and creation of new models.

The accuracy of automatic literature screening instruments varied widely in different algorithms and review topics [17]. The automatic literature screening systems can reach a sensitivity as high as 95%, despite at the expense of specificity, since reviewers try to include every publication relative to the topic of review. As the automatic systems may have a low specificity, it is also important to evaluate how much reviewing work the reviewers can save in the step of screening. We will not only assess the diagnostic accuracy of AI screening algorithms compared with human investigators, but also collect the information of work saved by AI algorithms in literature screening. Additionally, we plan to conduct subgroup analyses to identify potential factors that associate with the accuracy and efficacy of AI algorithms.

As far as we know, this will be the first systematic review to evaluate AI algorithms for automatic literature screening in evidence synthesis. Few systematic reviews have focused on the application of AI algorithms in medical practice. The literature search strategies in previous published systematic reviews rarely use specific algorithms as search terms. Most of them generally use words such as “artificial intelligence” and “machine learning” in strategies, which may lose the studies that only reported one specific algorithm. In order to include AI-related studies as much as possible, our search strategy contained all of the AI algorithms commonly used in the past 50 years, and it was reviewed by an expert in ML. The process of literature screening can be assessed under the framework of the diagnostic test. Findings from this proposed systematic review will provide a comprehensive and essential summary of the application of AI algorithms for automatic literature screening in evidence synthesis. The proposed systematic review may also help to improve and promote the automatic methods in evidence synthesis in the future by locating and identifying the potential weakness in the current AI models and methods.

## Supplementary Information

**Additional file 1: Supplementary Table 1**. Search strategy for PubMed.

## Data Availability

The datasets used and analysed during the current study are available from the corresponding author on reasonable request.

## Abbreviations

AI: Artificial intelligence
AUC: Area under the curve
DOR: Diagnostic odds ratio
GRADE: Grading of Recommendations, Assessment, Development and Evaluations
HSROC: Hierarchical summarised receiver operating characteristic curve
NLR: Negative likelihood ratio
NPV: Negative predictive value
PLR: Positive likelihood ratio
PPV: Positive predictive value
PRISMA-P: Preferred Reporting Items for Systematic Review and Meta-Analysis Protocols
QUADAS-2: Quality Assessment of Diagnostic Accuracy Studies
ROC: Receiver operating characteristic curve
SVM: Support vector machine
