# Supplementary material for: Automation of literature screening using machine learning in medical evidence synthesis: a diagnostic test accuracy systematic review protocol
Source: Syst Rev. 2022 Jan 15;11:11. doi: 10.1186/s13643-021-01881-5 (PMC8760775; doi:10.1186/s13643-021-01881-5)
Supplement: Supplementary file 1 — Additional file 1: Supplementary Table 1. Search strategy for PubMed. [file 13643_2021_1881_MOESM1_ESM.docx]

**Automation of Literature Screening using Machine Learning in Medical Evidence Synthesis: A Diagnostic Test Accuracy Systematic Review Protocol**

Supplementary Table 1. Search strategy for PubMed.

Here we provide the search strategy used in PubMed. The search strategy uses three core concepts: systematic review, literature screening, and artificial intelligence.

| PubMed Search terms |
| --- |
| 1 "medical evidence"[Title/Abstract] |
| 2 "PICO"[Title/Abstract] |
| 3 "PECODR"[Title/Abstract] |
| 4 "intervention arms"[Title/Abstract] |
| 5 "experimental methods"[Title/Abstract] |
| 6 "study design parameters"[Title/Abstract] |
| 7 "Patient oriented Evidence"[Title/Abstract] |
| 8 "eligibility criteria"[Title/Abstract] |
| 9 "evidence based medicine"[Title/Abstract] |
| 10 "clinically important elements"[Title/Abstract] |
| 11 "evidence based practice"[Title/Abstract] |
| 12 "evidence synthesis"[Title/Abstract] |
| 13 "results from clinical trials"[Title/Abstract] |
| 14 "research results"[Title/Abstract] |
| 15 "clinical evidence"[Title/Abstract] |
| 16 "Meta Analysis"[Title/Abstract] |
| 17 "Clinical Research"[Title/Abstract] |
| 18 "medical abstracts"[Title/Abstract] |
| 19 "clinical trial literature"[Title/Abstract] |
| 20 "clinical trial characteristics"[Title/Abstract] |
| 21 "clinical trial protocols"[Title/Abstract] |
| 22 "clinical practice guidelines"[Title/Abstract] |
| 23 "systematic review"[Title/Abstract] |
| 24 "systematic literature review"[Title/Abstract] |
| 25 "systematic scoping review"[Title/Abstract] |
| 26 "systematic prevalence review"[Title/Abstract] |
| 27 OR/1-26 |
| 28 "Artificial Intelligence"[Mesh] OR "Artificial Intelligence"[Title/Abstract] |
| 29 "natural language"[Title/Abstract] |
| 30 "language processing"[Title/Abstract] |
| 31 "Knowledge Acquisition"[Title/Abstract] |
| 32 "Knowledge Representation"[Title/Abstract] |
| 33 "Support Vector Machine"[Title/Abstract] OR "svm"[Title/Abstract] |
| 34 "Gaussian"[Title/Abstract] |
| 35 "Bayes"[Title/Abstract] OR "Bayesian"[Title/Abstract] |
| 36 "Cluster"[Title/Abstract] OR "Clustering"[Title/Abstract] |
| 37 "Hidden Markov"[Title/Abstract] |
| 38 "conditional random field"[Title/Abstract] |
| 39 "Random Forest"[Title/Abstract] |
| 40 "Graphical"[Title/Abstract] AND "model"[Title/Abstract] |
| 41 "Regression"[Title/Abstract] |
| 42 "feature engineering"[Title/Abstract] |
| 43 "zero-shot learning"[Title/Abstract] OR "few-shot learning"[Title/Abstract] |
| 44 "reinforcement learning"[Title/Abstract] |
| 45 "transfer learning"[Title/Abstract] |
| 46 unsupervised[Title/Abstract] OR supervised[Title/Abstract] OR semi-supervised[Title/Abstract] OR distant-supervised[Title/Abstract] OR self-supervised[Title/Abstract]) |
| 47 ("neural network"[Title/Abstract]) OR ("neural networks"[Title/Abstract]) OR (neural[Title/Abstract] AND algorithm*[Title/Abstract]) OR (neural[Title/Abstract] AND machine[Title/Abstract]) |
| 48 (network[Title/Abstract] AND algorithm*[Title/Abstract]) OR (network[Title/Abstract] AND machine[Title/Abstract]) |
| 49 (automatic[Title/Abstract] AND network[Title/Abstract]) OR (automatic[Title/Abstract] AND networks[Title/Abstract]) OR (automatic[Title/Abstract] AND algorithm*[Title/Abstract]) OR (automatic[Title/Abstract] AND model[Title/Abstract]) OR (automatic[Title/Abstract] AND models[Title/Abstract]) OR (automatic[Title/Abstract] AND machine[Title/Abstract]) OR (automatic[Title/Abstract] AND learning[Title/Abstract]) OR (automatic[Title/Abstract] AND method[Title/Abstract]) |
| 50 (learning[Title/Abstract] AND network[Title/Abstract]) OR (learning[Title/Abstract] AND networks[Title/Abstract]) OR (learning[Title/Abstract] AND algorithm*[Title/Abstract]) OR (learning[Title/Abstract] AND machine[Title/Abstract]) OR (learning[Title/Abstract] AND method[Title/Abstract]) |
| 51 (deep[Title/Abstract] AND network[Title/Abstract]) OR (deep[Title/Abstract] AND networks[Title/Abstract]) OR (deep[Title/Abstract] AND algorithm*[Title/Abstract]) OR (deep[Title/Abstract] AND model[Title/Abstract]) OR (deep[Title/Abstract] AND models[Title/Abstract]) OR (deep[Title/Abstract] AND machine[Title/Abstract]) OR (deep[Title/Abstract] AND learning[Title/Abstract]) |
| 52 OR/28-51 |
| 53 "extract*"[Title] |
| 54 "classif*"[Title] |
| 55 "identif*"[Title] |
| 56 "retriev*"[Title] |
| 57 "detect*"[Title] |
| 58 "judg*"[Title] |
| 59 "determin*"[Title] |
| 60 "decid*"[Title] |
| 61 "sort*"[Title] |
| 62 "infer*"[Title] |
| 63 "interpret*"[Title] |
| 64 "includ*"[Title] |
| 65 "exclud*"[Title] |
| 66 "screen*"[Title] |
| 67 "filter"[Title] OR "filtering"[Title] |
| 68 "select*"[Title]  69 "sift*"[Title] |
| 70 OR/53-69 |
| 71 27 AND 52 AND 70 |
